# Web-based interventions for weight loss and weight maintenance among rural midlife and older women: protocol for a randomized controlled trial

**DOI:** 10.1186/1471-2458-11-521

**Published:** 2011-06-30

**Authors:** Patricia A Hageman, Carol H Pullen, Melody Hertzog, Linda S Boeckner, Susan Noble Walker

**Affiliations:** 1 Physical Therapy Education, School of Allied Health Professions, College of Medicine, University of Nebraska Medical Center, Omaha, Nebraska, USA; 2 College of Nursing, University of Nebraska Medical Center, Omaha, NE, USA; 3 College of Nursing, University of Nebraska Medical Center, Lincoln, NE, USA; 4 Panhandle Research and Extension, University of Nebraska-Lincoln, Scottsbluff, Nebraska, USA

## Abstract

**Background:**

Weight loss is challenging and maintenance of weight loss is problematic among midlife and older rural women. Finding effective interventions using innovative delivery methods that can reach underserved and vulnerable populations of overweight and obese rural women is a public health challenge.

**Methods/Design:**

This Women Weigh-In for Wellness (The WWW study) randomized-controlled trial is designed to compare the effectiveness of theory-based behavior-change interventions using (1) website only, (2) website with peer-led support, or (3) website with professional email-counseling to facilitate initial weight loss (baseline to 6 months), guided continuing weight loss and maintenance (7-18 months) and self-directed weight maintenance (19-30 months) among rural women ages 45-69 with a BMI of 28-45. Recruitment efforts using local media will target 306 rural women who live within driving distance of a community college site where assessments will be conducted at baseline, 3, 6, 12, 18, 24 and 30 months by research nurses blinded to group assignments. Primary outcomes include changes in body weight, % weight loss, and eating and activity behavioral and biomarkers from baseline to each subsequent assessment. Secondary outcomes will be percentage of women achieving at least 5% and 10% weight loss without regain from baseline to 6, 18, and 30 months and achieving healthy eating and activity targets. Data analysis will use generalized estimating equations to analyze average change across groups and group differences in proportion of participants achieving target weight loss levels.

**Discussion:**

The Women Weigh-In for Wellness study compares innovative web-based alternatives for providing lifestyle behavior-change interventions for promoting weight loss and weight maintenance among rural women. If effective, such interventions would offer potential for reducing overweight and obesity among a vulnerable, hard-to-reach, population of rural women.

**Trial Registration:**

ClinicalTrials.gov: NCT01307644

## Background

With obesity now considered an epidemic, multiple national initiatives are emphasizing the need to assist Americans in balancing healthful eating with regular physical activity [1-5]. The Strategic Plan for NIH Obesity Research emphasizes the need to include understudied rural populations and to design and test interventions using behavioral approaches to lifestyle change [6]. The Nurse's Health Study found evidence that adiposity in midlife is strongly correlated to a reduction in healthy outcomes among women when they reach age 70 [7].

Approximately two-thirds of midlife and older women in the USA are overweight or obese, with greater percentages (75%) of overweight and obesity reported among rural than among urban women [8]. Rural isolation may challenge rural women's development of healthy lifestyle patterns, as rural women may have differing eating patterns and increased barriers to physical activity [9,10]. While rural women have significantly higher rates of sedentary behavior and consumption of high fat diets, they are less likely to receive counseling in diet and activity or to be offered preventive measures than their urban counterparts [8,11]. This issue increases the importance of finding innovative ways to offer health and preventive services for this group.

Interactive web-based interventions for weight loss and maintenance have been shown to be effective in younger and middle-aged adults [12-16]. The proposed study is unique in targeting peri- and post-menopausal rural women whose health risks become magnified if they are overweight or obese [17,18]. Internet delivery of a behavior-change intervention to achieve weight loss and maintenance may be well received as middle aged and older women increasingly use the Internet as a supplemental source for health information [19,20].

Internet access is growing in rural communities, though rural residents may have fewer choices about the way they access the Internet and may have narrower bandwidth than urban residents [21]. Access to the Internet is available in 81% of households in the Midwestern state where this intervention is to be implemented [22]. Two separate pilot studies demonstrated that it was feasible to implement a behavior-change intervention using web delivery in midlife and older rural women [23,24]. We found that the majority of rural women enrolled in a web-based weight loss study were able to retrieve weight loss information from our intervention web-site and most participants said the web-based messages were helpful to them in trying to lose weight [24].

Studies using lifestyle modification for weight loss and weight maintenance suggest that 16 to 26 weeks is needed to achieve a loss of 5% to 10% of body weight, which is the percentage loss associated with health benefits [25-27]. The duration of weight maintenance interventions has been correlated with the likelihood of continued weight maintenance [28,29] as weight regain occurs within 1 to 2 years for most people. Based on this, we plan for a three phase intervention approach with phase 1 targeting weight loss (baseline to 6 months), phase 2 targeting guided continuing weight loss and maintenance (7-18 months), and phase 3 targeting self-directed weight maintenance (19-30 months). In prior work, we discovered that rural women were willing to participate in and remain committed to a 12-month behavior-change intervention using mailed newsletters and a 12-month follow up, with the attrition rate being 4.5% over 24 months [30]. Those women successfully adopted and maintained healthy eating and activity behaviors [30].

Website interventions have weight loss results as successful as in-person interventions, if not better [31,32]. A review of 21 technology-based studies identified self monitoring, counselor feedback, social support, use of a structured program and use of an individually tailored program, to be keys to short-term weight loss, with implications that these components may be helpful for long-term weight loss studies such as our study [33]. The major challenge with implementing web-based interventions is keeping participants engaged over the length of the study. Successful strategies have included an in-depth participant orientation [34] and planned reminder systems [31,34], methods that we will include in this study.

To achieve long-term weight maintenance, prolonged frequent in-person contacts are recommended [35]. Frequent contacts are typical of community weight loss programs; yet, such programming may not be feasible for rural women. One of the most common uses of the Internet is the exchange of information and support via peers, so using the Internet for peer-led support or professional email-counseling may be a viable option for providing frequent personal contacts necessary for long term weight maintenance [36]. Peer-led support groups in face-to-face formats were found to be effective in treatment of obesity [37,38]; however, no robust evidence was found for the effectiveness of peer-to-peer groups delivered by the Internet because some studies provided peer support by health professionals and other studies used complex interventions where it was not possible to separate the effects of the peer component [13,15]. In our 3-month pilot weight-loss study of rural women ages 50-69, we found that access to an interactive website plus a peer-led asynchronous support group enhanced weight loss [24].

Lifestyle modification interventions that provided continued contact with professional counselors were also found to be effective in achieving long-term weight loss maintenance [39]. Professional counseling for weight loss has been conducted using email. In two separate studies of overweight hospital employees and individuals at risk for type II diabetes, those in Internet education plus email professional counseling group lost significantly more weight than those in an Internet education only group after 6 months [12,15]. Varying results have been reported in achieving and maintaining weight loss in studies that incorporated weight loss counseling via email involving mostly midlife Caucasian women [13,15].

The literature is not clear whether web-based peer-support or professional email-counseling would be more advantageous methods for providing ongoing support for weight loss and maintenance. If peer-led online support groups are at least as successful in promoting weight loss and maintenance as individual professional email-counseling, the financial savings could be considerable.

This paper describes the protocol for a randomized controlled trial that aims to promote healthy eating and activity in order to achieve a targeted weight loss of 5% to 10% and weight maintenance among rural women ages 45-69 with a body mass index (BMI) of 28-45. To our knowledge, this study is the first to use a theory-based behavior-change intervention targeting midlife and rural women with an interactive web-based intervention for weight loss and weight maintenance, or to compare peer-led support to professional counseling.

### Aims and hypothesis

The primary aim is to compare the effectiveness of an interactive website only, interactive website plus a peer-led online support group, and interactive website plus professional weight loss counseling via email in facilitating initial weight loss (baseline to 6 months), guided continuing weight loss and maintenance (7-18 months) and self-directed weight maintenance (19-30 months). The secondary aim is to compare the 3 groups in improving healthy eating and activity behavioral and biomarkers.

We hypothesize that the groups who receive either peer-led online support or professional weight loss counseling will achieve better weight loss and weight maintenance outcomes than the group who receives interactive website only. There is insufficient evidence to hypothesize the relative effectiveness of peer-led online support and professional weight loss counseling.

## Methods/Design

### Ethics approval

The Institutional Review Board of the University of Nebraska Medical Center approved this study (approval number 23710-FB). Using the standardized and accepted protocol, written informed consent will be obtained from all participants prior to their enrollment.

### Study design

**Figure 1:**
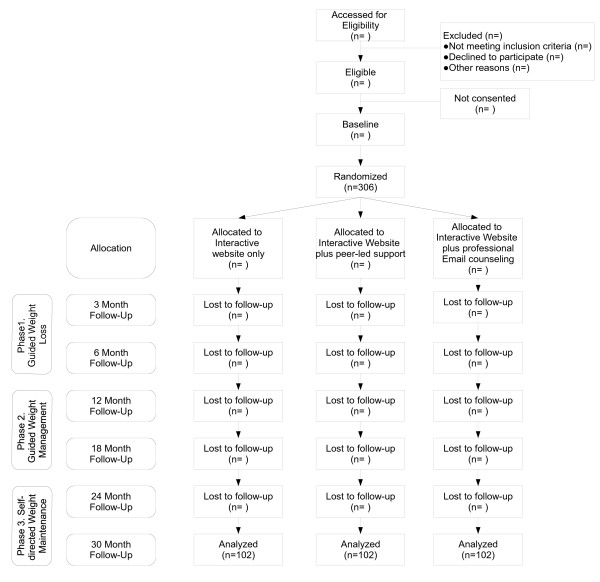
**CONSORT Participant Flowchart for the Women Weigh-In for Wellness Study**.

The study was designed as a randomized controlled trial and is summarized in Figure 1. Primary outcomes will be change in weight biomarkers and healthy eating and activity behavioral and biomarkers from baseline to subsequent assessments of 3, 6, 12, 18, 24, and 30 months. Secondary outcomes will be the percentage of women achieving at least 5% and 10% weight loss from baseline without regain, as well as achieving healthy eating and activity criterion standards at time points that correspond with the intervention phases (6, 18, 30 months). This study will be conducted in accordance with the Consolidated Standards of Reporting Trials (CONSORT) guidelines [40].

### Sample size

Sample size needed for the study was estimated for a test of group difference in response averaged across time using generalized estimating equations [41]. Calculations assumed a group mean difference of .45 standard deviations based on median values on these outcomes in [12,15] and our pilot work, two measurement times per phase, within-subject correlation ≤ .5, and α of .017 (Bonferroni adjustment for three pairwise comparisons). For the most conservative pairwise test that will be conducted, the two-sided comparison of the groups receiving counseling, 76 participants will be needed in each group. A total sample size of 306 will be recruited to allow for attrition of 25%, consistent with that experienced in both published weight loss studies and our own pilot work.

### Inclusion criteria

Eligible participants will be women ages 45-69, with a body mass index of 28 to 45, who are residents in one of 10 rural counties in a midwestern state in the USA [26]. The potential pool of women classified as minority (predominantly of Hispanic origin) is < 1.6%. Other inclusion criteria include being able to: speak and read English, communicate over the phone, use a computer with minimal assistance to complete Internet surveys, walk without an assistive device or oxygen, have access to the Internet and a DVD player. Eligible women will be included if they answer "no" to all questions on the Physical Activity Readiness Questionnaire (PAR-Q) or obtain medical clearance from their physician to participate [42]. Women who have a BMI from 40-45 or who are diagnosed with Type II diabetes without requirements for insulin will be required to obtain medical clearance.

### Exclusion criteria

Women will be excluded if they are overweight with a BMI of 25-27.99 because they would reach normal weight with less than 5% loss, or if they have a BMI over 45, and thus may require a different approach to weight loss [26]. Women also will be excluded if they have a diagnosis of Type 1 diabetes or have a diagnosis with Type 2 diabetes and require insulin. Other exclusionary criteria include having a history of 10% or greater weight loss in the last six months, being enrolled currently in a weight loss management program or a formal program of cardiac or physical rehabilitation, taking medications that affect weight loss or weight gain, participating in current cancer treatment, or having any other physical or medical restrictions that would preclude following the recommendations for moderate physical activity and health eating.

### Participant recruitment and enrollment

A total of 306 women will be recruited over a 10 month period, during summer 2011 through spring 2012, from the 10 counties within 50 miles of the local community college where the assessments will be completed. Planned recruitment methods include speaking at community events and targeting potential participants using newspaper, radio and flyers posted in the community. Individual contacts will be made with local physicians asking them to refer overweight/obese women to the study. The recruiter will work closely with the county health department personnel who serve mostly low income and/or Hispanic populations to recruit persons with Hispanic backgrounds and persons of lower socioeconomic levels.

The recruiter will conduct a preliminary screening interview with potential participants to establish eligibility for the study and provide information for the informed consent process. The recruiter will provide contact information to the research nurses at the assessment office for each eligible woman who agrees to participate. These nurses will contact the women to verify if physician clearance was obtained, will schedule appointments to complete the consent process, and will begin baseline assessment if eligible.

### Baseline assessment

The baseline assessment will be conducted over 2 visits scheduled within 3 weeks. All other assessments will require one visit only. The research nurses will orient women to access the Women Weigh-In for Wellness website and instruct them on use of an accelerometer and a pedometer.

The women will be randomly assigned to one of 3 intervention groups upon completion of baseline assessment visit 2, using an allocation schedule previously created by the project statistician, who will not have any contact with the women during the trial. Each woman will receive notice of her randomization electronically at the first login after completing baseline visit 2. The research nurse will remain blinded to group assignment throughout the study.

### Interventions

#### Conceptual framework and background

The Health Promotion Model, with theoretical underpinnings in Social Cognitive Theory, was selected as a framework for the research to explain and modify the adoption and maintenance of healthy eating and activity behaviors needed for weight loss and maintenance in this study [43]. The Health Promotion Model emphasizes the active role of an individual in self-initiation and maintenance of health behaviors. Constructs selected from the model for use in the intervention design of this study are those for which there is empirical evidence supporting predictive validity for modifying behavior, including four behavior-specific cognitions (perceived benefits of action, barriers to action, self-efficacy and interpersonal influences) as well as commitment to a plan of action through goal setting. Prior work supports the association of these constructs with eating and activity behaviors among midlife and older rural women [44].

#### Intervention groups

All three theory-based interactive web-delivery intervention groups will receive the same content which provides a lifestyle change plan to accomplish their individual pound targets for a minimum of 5% and preferably 10% weight loss. The weight loss and maintenance approach will follow recommendations from the 2010 Dietary Guidelines for Americans [5], the 2008 Physical Activity Guidelines for Americans [3] and Healthy People 2010 [4]. The plan goals will be to achieve a weight loss of 1-2 pounds per week in the first 6 months by reducing caloric and fat intakes and gradually increasing physical activity to recommended levels. Caloric intakes will be reduced by 500-1000 kilocalories from baseline caloric needs, with a minimum caloric level established at 1200 kilocalories. Sedentary physical activity levels were assumed in calculating caloric needs.

To monitor and enhance the reliability and validity of our behavioral interventions, we developed an intervention fidelity plan described in Table 1, that addresses the design of the study, training of the interventionists, delivery of the intervention, receipt of the treatment by the participant, and enactment that ensures that the participant performs the skills and strategies as intended, based on Resnick's comprehensive model [45].

**Table 1:** Women Weigh-In for Wellness Intervention Fidelity Plan.

Study Component	Fidelity Plan
Design	1. Ensure same intervention dose within conditions
	• Fixed content in internet web-messaging
	• Feedback about weight logs and eating activity goals provided at fixed intervals
	• Professional weight loss counseling provided using fixed length, frequency and number of contact sessions
	• Peer support group provided using fixed frequency and fixed content of discussion topics posted
	• Any deviations from protocol recorded
	2. Ensure equivalent dose across conditions
	• Web-messaging content identical across intervention groups
	• Frequency of additional contact for women in professional counseling email group and peer-led discussion group is similar
	3. Plan for implementation setbacks
	• Train back-up providers for professional weight loss counselor and peer support leader positions

Training	1. Training of all personnel by investigators initially and at specified intervals
	2. Guidelines for content of email messages and discussion board postings established

Intervention Delivery	1. Investigator-prepared guide for content/concepts in newsletters and hot topics
	2. Investigator oversight of newsletters and hot topics before website posting
	3. For professional weight loss counseling via email
	• Emails copied to and monitored by investigators
	• Email messages sent and received printed and filed weekly
	4. For peer-led online support group
	• List of topics prepared have oversight by investigators prior to testing
	• Project coordinator and/or investigators monitors discussion board weekly
	• Record of discussions printed and filed weekly

Intervention Receipt	1. Monitoring of all participants
	• Goal-setting forms
	• Weight logs
	• Weekly hits by each participant
	2. Monitoring of participants in peer-led online support group
	• Log-in
	• Postings
	3. Monitoring of professional weight loss counseling via email
	• Return receipt used to monitor opening of email messages
	• Replies from participants printed and filed

Enactment	1. Monitoring of behavioral skills and cognitive strategies of participants
	• Attainment of goals
	• Assessments of weight, and eating and activity behavioral and biomarkers
	• Assessments of cognitive-perceptual influences at 6,18, and 30 months

#### Phase 1 guided weight loss

New messages will be posted on the website each week for the first 6 months, and will remain available throughout the study. The women will receive an email with a preview of the content and a link to the site each time new content is posted. The messages will be targeted to midlife and older rural women and both an educational and a behavioral change approach will be used in the design of the messages. An eating and activity plan will provide content relevant to weight loss. Behavioral approaches will include self-monitoring tools, goal setting, individualized feedback regarding assessment results, relapse techniques, and motivational messages related to benefits of and barriers to action, self-efficacy, and interpersonal support. Interactive features such as quizzes, logs, and short games will be included as well as a resource area. Since self-monitoring is an important strategy in weight loss, women will be requested to post their eating and activity behaviors daily, and weights and goals weekly. Graphs of eating, activity, and body weight will be generated from the women's log entries to provide visual feedback to each woman about her progress.

A trained peer leader will facilitate an asynchronous discussion with those women enrolled in the interactive web-site plus peer counseling group. The peer leader will post a message once a week and participate in the discussion with the women about topics of interest relevant to the weekly messages. Because recruitment will occur over 10 months, the women will be placed in small discussion groupings based upon their time of enrollment. For intervention fidelity, the peer-led support discussion group will be monitored weekly by one of the investigators, and each woman's log-ins and postings will be tracked.

The interactive web-site plus professional email-counseling group will have their eating, activity and weight logs and goals on the website monitored by a professional counselor experienced in healthy eating and activity to accomplish weight loss. The counselor will send email feedback once a week using an email process following the 5A's Model for Behavioral Counseling (assess, advise, agree, assist and arrange) [46] that was adapted for this study. The women can email questions for the counselor to answer. The counselor will blind copy an assigned investigator on all emails and a return receipt will be issued to monitor women's opening of emails.

#### Phase 2 guided continuing weight loss/maintenance

The purpose of phase 2 will be to promote continued weight loss for those women who have not lost at least 10% of their baseline weight and to promote maintenance of that loss for those women achieving a 10% weight loss. The activity target will increase to a minimum of 60-90 minutes per day consistent with current guidelines [47,48].

At the 6-month assessment, women in all groups will be given an investigator-produced DVD [Banding Together for Strength] with 2 latex-free resistive bands to assist them in starting or continuing resistance training in the convenience of their own homes. The DVD uses age appropriate exercises led by age-related role models in order to enhance self-efficacy for resistance training. The women will have access to all Internet messages received in phase 1 for their review. Phase 2 new messages will focus on hot topics in the form of a news brief posted every week in months 7-9, every 2 weeks in months 10-13, and monthly in months 14-18. Content will focus on timely articles based on the literature with an emphasis on women taking more responsibility for their weight and moving toward permanent lifestyle change. Women will be asked to continue posting weights weekly and goals every two weeks.

The peer-led support group will receive a posted message for the discussion board from the peer leader weekly in months 7-9, every 2 weeks in months 10-13 and monthly in months 14-18. The peer leader will continue to participate in weekly discussions and will encourage the women to continue to discuss issues related to weight loss.

The group receiving professional email-counseling will have their postings of weight, activity, food, and goals reviewed weekly in months 7-9, every two weeks in months 10-13 and monthly in months 14-18. The counselor will send one reminder email each time that a woman has not posted as requested and the counselor will continue to respond to email questions from women throughout phase 2.

#### Phase 3 self directed weight maintenance

The purpose of phase 3 is to move women toward independence. Less support will be given, but sites to post eating, activity, goals and weight will be available. The target will be prevention of weight regain, with encouragement provided to the women to maintain their 5% to 10% weight loss by using the skills they have developed. The goal is for women to have made permanent lifestyle changes by the end of phase 3.

All three interactive-website intervention groups will have access to a content library on the website with hyperlinks to web-based resources intended to foster a reliable method of self-directed learning. Carefully selected articles related to healthy eating, activity, weight loss and weight maintenance will be organized into subject folders on the website for ease of participant access. Women will be encouraged to post weekly weight, and set/revise goals every two weeks.

Women assigned to the website plus peer-lead support group will have access to the discussion area but there will be no participation or postings by the peer leader.

Women assigned to the website plus professional email-counseling group will receive responses from the professional counselor to individual women's emailed questions; however, the counselor will not be providing feedback on the weight, eating, activity or goal setting logs.

### Data collection

Assessment of all variables will occur at baseline, 6 months, 18 months, and 30 months which correspond with the study phases. Women will be asked to attend a short assessment session at 3 months, 12 months, and 24 months which will include all measures except the Health Promotion Model cognitive-perceptual variables, blood glucose and lipids, self-reported diet, accelerometer data and estimated cardiorepiratory fitness via the 400-meter walk test. Established reliable and valid measures will be used for all assessments.

Questions pertaining to general demographic information and health history will be asked. The Patient-Reported Outcomes Measurement System (PROMIS-29 Profile v1.0) will be administered to obtain subscales of physical function, anxiety, depression, fatigue, sleep disturbance, social role, pain interference and intensity [49].

#### Blood pressure and resting heart rate

Resting heart rate (using the radial pulse for 60 seconds) and blood pressure will be measured following 5 minutes of quiet sitting following standardized methods [50]. At each visit, at least two blood pressure measurements separated by at least 30 seconds will be obtained. Systolic blood pressure is the appearance of the first Korotokoff sound. Diastolic blood pressure is the disappearance of Korotokoff sounds. Both systolic and diastolic blood pressure will be recorded as the mean of the two measurements that were within 5 mmHg. Participants will be asked to avoid caffeine, exercise and smoking for at least 30 minutes prior to an assessment.

#### Eating

The Web version of the 1998 Block Health Habit and History Questionnaire will be used to measure the usual self-selected diets of individuals over a period of time [51]. This survey asks for the frequency of consumption of particular food items consumed in the last 3 months. It provides information about daily caloric intake, % of dietary intake by categories (protein, carbohydrate, % fat, % saturated fat), intake of daily servings (fruits, vegetables, whole grain, low fat dairy) and daily mineral consumption.

#### Physical activity

The Behavioral Risk Factor Surveillance System (BRFSS): Physical Activity Module consisting of a 7-item self-report instrument will be used to determine the women's participation in moderate or vigorous activity by days per week and minutes per day [52]. In addition, the BRFSS allows for categorization of participation by physical activity as recommended, insufficient or inactive as defined by the United States Department of Health and Human Services [53]. Using a format similar to the BRFSS, two items from the Women's Exercise Injuries: Incident and Risk Factors study will be added to capture self-reported minutes per day and days per week spent in strengthening activity and walking [54].

Time spent weekly at each study phase (baseline, 6-month, 18 month and 30-month) in light, moderate and vigorous intensity physical activity will be measured objectively by the triaxial Actigraph accelerometer (GT3X) using methods consistent with the publicly available SAS code developed to process the 2003-2004 NHANES accelerometer-determined physical activity data [55,56]. Individuals will be asked to wear the Actigraph for 24 hours a day, except when showering, and to record the time spent during sleeping for comparison to the Actigraph data.

The 400-meter walk test, shown to have excellent reproducibility and strong associations with estimated and measured maximum oxygen consumption in midlife and older women, will be used to provide an estimate of cardiorespiratory fitness [57]. Women will be asked to walk a 400-meter course at a brisk pace with total walk time recorded, and heart rate recorded in beats per minute recorded prior to and immediately following the walk. To minimize learning effects at baseline, the best walk times of two trials completed on separate days will be recorded. Lower body strength will be assessed using the 10-repetition timed chair stand test that requires completion of 10 full stands from a sitting position as quickly as possible while timed in seconds [58]. Upper body strength will be measured using the timed arm curl test which involves counting the number of times a woman can curl a 5-pound hand weight through a full range of motion in 30 seconds [59].

#### Biomarkers affected by activity and eating

The Tanita Model [TBF-215, Tanita Corporation of America, Inc., 2625 S. Clearbrook Dr., Arlington Heights, IL 60005-9824] will be used to measure height, weight and percent body fat following the manufacturer's instructions. Women will be asked to fast within 4 hours of the test, not exercise intensively within 12 hours of the test, avoid alcohol 48 hours before testing and to void the bladder within 30 minutes prior to the test, as this bioelectrical impedance analysis system measure is sensitive to hydration status [60,61].

BMI will be calculated as weight in kilograms divided by height in meters-squared [26]. Waist circumference will be measured by placing a tape in a horizontal plane around the abdomen at the level of the iliac crest at the end of expiration. Hip circumference will be measured by placing a tape in a horizontal plane at the widest element of the hips between the greater trochanters and the gluteal fold. These anthropometric procedures will require a snug tape that is parallel to the floor but is held without skin compression with the average of two trials recorded [26].

Blood specimens will be drawn after a 12-hour fast to determine total cholesterol, low-density lipoprotein (LCL-C), high-density lipoprotein (HDL-C), triglycerides and fasting glucose following standardized protocols.

#### Measurement of behavioral determinants

Women will complete validated surveys to assess their perceptions of benefits and barriers, interpersonal support and self-efficacy for healthy eating and activity. The instruments will include the Exercise Benefits/Barriers Scales [62]; Healthy Eating Benefits/Barriers Scales [44]; Self-efficacy for Eating Habits Scale; Self-Efficacy for Exercise Habits Scale [63]; and Family Support for Exercise Habits Scale and Friend Support for Exercise Habits Scale; and Family Support for Healthy Eating Habits Scale and Friend Support for Healthy Eating Habits Scale [64].

#### Process evaluation

Process evaluation forms without identifiers, but color-coded for group designation, will be mailed to participants with a postage paid envelope for return at 6, 18, and 30 months to determine the degree to which the program was implemented as planned. Questions are designed to assess the women's recall of the intervention, the extent to which they participated and how helpful the intervention was in assisting them to make change. Website use will be collected from server log files. In addition, at 6 and 18 months, we will conduct telephone interviews with a random sample of approximately 10 participants from each intervention group, seeking representation from those women who were successful with weight loss of at least 5% and those who have not been successful with this weight loss. A similar approach will be followed at the end of the study, 30 months after baseline, using a face-to-face focus group format for each of the intervention groups to be conducted at the rural assessment site.

#### Data analysis

The primary analysis will focus on each phase separately, testing planned pairwise comparisons of the intervention groups over time using generalized estimating equations (GEE). The primary end point will be average change in outcome from the beginning of each phase. GEE also will be used to test whether the groups differ over time in the proportion of women meeting 5% and 10% weight loss goals. Secondary analyses will include a trend analysis of differences in the pattern of change across the entire 30 months of the study. All primary analyses will be consistent with the intent-to-treat principle and imputation of missing values may be undertaken, depending upon the extent and pattern of missing data.

## Discussion

Providing an effective intervention for behavior-change to promote weight loss and weight maintenance among midlife and older rural women is needed, as these women have a high prevalence of overweight and obesity. These theory-based web-delivery interventions offer innovative approaches to offer weight loss and maintenance counseling to this hard-to-reach vulnerable group of rural women.

The intervention has merit in investigating the effectiveness of distance delivery methods to encourage multiple behavior-changes in the areas of healthy eating and activity for weight loss. A strength of this study is that it examines a comprehensive approach for behavior-change by incorporating the Health Promotion Model's concepts of behavior-specific cognitions (barriers to and benefits of action, interpersonal support and self-efficacy for healthy eating and activity), self-monitoring (home weight scales, pedometers and logs), individualized feedback, and cues to action (goal setting) as part of the intervention. In addition, the study design provides an extended period for weight maintenance, which has been linked with continued weight maintenance in other populations.

Limitations are acknowledged as self-reported measures of activity and eating may be biased by social desirability of response, although self-reports will be validated by assessments of biomarkers known to change in conjunction with these behaviors. As it is not feasible to randomly sample women who are overweight or obese, the sample of convenience may limit the generalizability of the results although the sample will be representative of midlife and older rural underserved women and the women will be randomized to intervention group. The lack of minorities in the areas from which the women will be recruited will limit the generalizability of results to the general population. We anticipate that the sample is biased in favor of those who have a stronger interest in changing their health behaviors and of those who have Internet access. Increased societal attention towards addressing overweight and obesity that permeates the media may influence behavior-change concurrently with our intervention.

This research will implement a distance model for delivery of lifestyle modification and information to provide easily accessible lifestyle guidance to a hard-to-reach clientele. If effective, these interventions may also provide benefit to over-worked and under-reimbursed rural providers who may not have the time to deliver lifestyle modification programs. If any of the interventions are found effective, follow up testing for pre-packaged programs for the public offered through settings such as public health departments, primary care offices, or health education settings may be appropriate.

## Competing interests

The authors declare funding received from the National Institute of Nursing Research, National Institutes of Health grant (1R01NR010589) for the article-processing charge.

## Authors' contributions

All authors conceived and designed the study, and obtained ethics approval. CHP provided leadership as primary investigator to this project and all authors were responsible for the acquisition of funding for this project. MH conducted the sample size calculation and created the plan for statistical analysis. PAH and CHP drafted the manuscript and all authors contributed to its revision. All authors read and approved the final manuscript.

## Pre-publication history

The pre-publication history for this paper can be accessed here:

http://www.biomedcentral.com/1471-2458/11/521/prepub
